# Respiratory failure caused by impending tension pneumothorax after extrapleural pneumonectomy: a case report

**DOI:** 10.1186/s40981-018-0184-z

**Published:** 2018-06-06

**Authors:** Sonoko Sakuraba, Takeshi Omae, Izumi Kawagoe, Keito Koh, Eiichi Inada

**Affiliations:** 1 Department of Anesthesiology and Pain Clinic, Juntendo University Shizuoka Hospital, 1129 Nagaoka, Izunokuni, Shizuoka 410-2295 Japan; 2 Department of Anesthesiology and Pain Medicine, School of Medicine, Juntendo University, Tokyo, Japan

**Keywords:** Extrapleural pneumonectomy, Pneumothorax, Respiratory failure, Cardiac herniation

## Abstract

**Background:**

Cardiac herniation is a serious postoperative complication of extrapleural pneumonectomy (EPP) and is reportedly preventable by reducing the suction pressure of the chest drain.

**Case presentation:**

We describe a patient in whom respiratory failure, which was caused by impending tension pneumothorax after EPP, was successfully treated via normal suction pressure of the chest drain. A lower suction pressure (− 7 cmH_2_O) was chosen as an alternative to the setting typically used for postoperative drainage (− 15 cmH_2_O). As a result, the wound in the chest wall functioned as an antireflux check valve, leading to the development of impending tension pneumothorax.

**Conclusions:**

Impending tension pneumothorax presents with an abnormal elevation of intrapleural pressure on the affected side. This phenomenon can be effectively treated by increasing the suction pressure in the chest drain.

## Background

Extrapleural pneumonectomy (EPP) is a surgical procedure involving en bloc resection of the affected lung and a part of the diaphragm and pericardium. Because of its high invasiveness, several complications have been reported in association with the procedure. Major complications of EPP include atrial fibrillation and other forms of arrhythmia, as well as heart failure caused by increased right ventricular strain related to the absence of one lung [1–3]. Complications requiring surgery have been reported, including cardiac herniation, cardiac tamponade, bronchopleural fistula, and diaphragmatic hernia [4]. The present report describes our experience with a patient who developed impending tension pneumothorax on the affected side after EPP, followed by hypercarbia associated with compression of the unaffected lung.

## Case presentation

A 33-year-old man (height, 176 cm; weight, 71 kg) visited our hospital with a chief complaint of respiratory discomfort. The patient reported a history of smoking (20 cigarettes per day) between 20 and 31 years of age; otherwise, his medical and family histories were unremarkable. Computed tomography detected pleural effusion and an anterior mediastinal tumor. A detailed examination led to the diagnosis of thymoma (stage IVb: pleural dissemination involving the superior vena cava [SVC] and brachiocephalic vein). The patient exhibited a partial response to radiation therapy and chemotherapy; thus, the implementation of right EPP and partial replacement of the SVC were scheduled with the aim of achieving cytoreduction by removing as much of the thymoma as possible.

Preoperative respiratory function test results were as follows: vital capacity (VC), 5.51 L; %VC, 113.6%; forced expiratory volume in 1 s (FEV_1_), 4.6 L; and FEV 1.0%, 85.5%. Blood gas testing revealed a pH of 7.422; partial pressure of carbon dioxide (PaCO_2_), 40 mmHg; partial pressure of oxygen (PaO_2_), 96.9 mmHg; and bicarbonate (HCO_3_^−^), 25.6 mmol/L. No abnormalities were observed in preoperative cardiac function. Chest radiography revealed a mediastinal tumor shadow, but no abnormalities were detected in the lung fields.

On arrival to the operating room, the epidural catheter was inserted at the level of thoracic vertebrae 7/8 successfully. General anesthesia was induced by using remifentanil (1.0 μg/kg/min), rocuronium (40 mg), and propofol (target-controlled infusion [TCI], 3.0 μg/mL). The airway was secured using a 37-Fr left-sided double lumen tube (Smiths Medical, Minneapolis, MN, USA), which was inserted at a point above the bifurcation of the trachea via a bronchoscope. Anesthesia was maintained using propofol (TCI 3.0 μg/mL) and remifentanil (0.4–0.8 μg/kg/min), under 40–100% oxygen. Monitoring tools included a five-lead ECG system, pulse oximeter, and expired gas monitor. Bispectral index (BIS™, Medtronic PLC, Dublin, Ireland) was used to monitor the depth of anesthesia. Hemodynamic monitoring was performed via direct measurement of arterial pressure and measurement of central venous pressure. The chest lining was cut, the right phrenic nerve was separated, and the right diaphragm was removed. The left brachiocephalic vein was bypassed to the right atrial appendage, and the right brachiocephalic vein was bypassed to the SVC. The portion of the SVC with suspected residual tumor was dissected. After the lung hilum was treated, both the tumor and the right lung were removed; some injury to the trachea was repaired. The right diaphragm and pericardium were reconstructed using a GORE-TEX® sheet (W.L. Gore and Associates, Newark, NJ, USA). The procedure was performed while visually observing the left lung. As the anterior junction line was maintained, a left-sided chest drain was not inserted. The initial dose of 0.2% ropivacaine (5 mL) was injected into the epidural space when the chest incision was closed. Then, a continuous infusion of 0.2% ropivacaine (5 mL/h) without narcotics was initiated for postoperative analgesia. No abnormalities were observed in terms of intraoperative ECG and hemodynamics. The duration of anesthesia was 10 h and 12 min; the operative time was 8 h and 5 min. The infusion volume was 3550 mL, blood loss volume was 1540 g, and urine volume was 2120 mL.

**Fig. 1:**
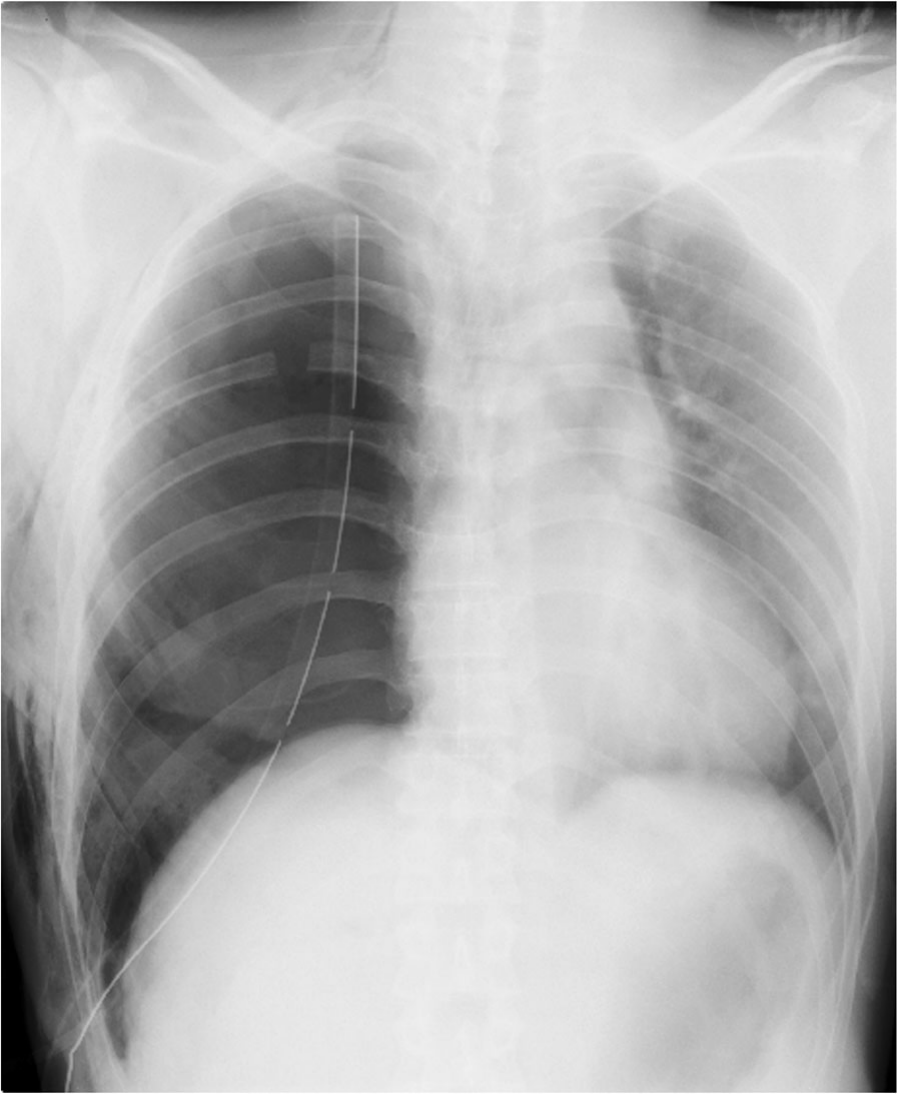
Chest radiograph before the elevation of the chest drain’s suction pressure from 7 to 15 cmH_2_O. This reveals mediastinal shift that may have been attributable to tension pneumothorax

**Fig. 2:**
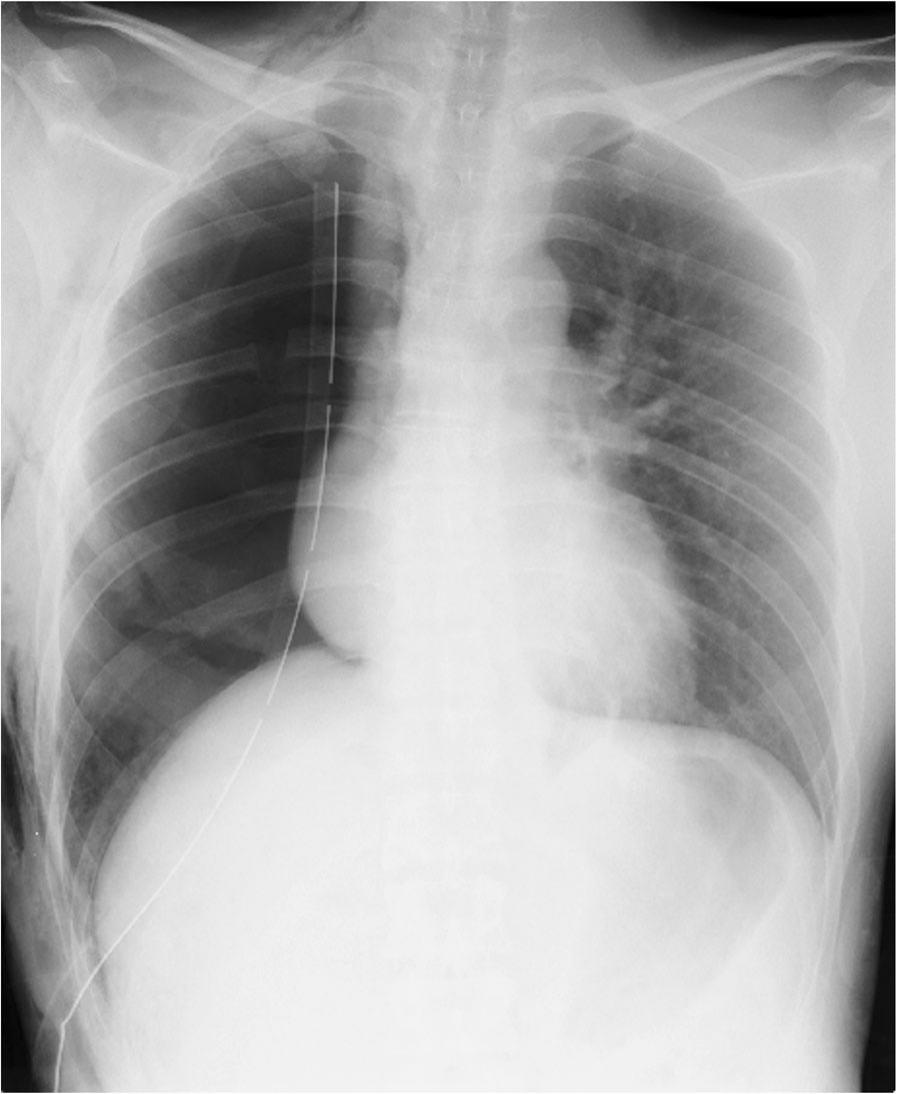
Chest radiograph after the elevation of the chest drain’s suction pressure from 7 to 15 cmH_2_O. This reveals improvement of the mediastinal shift and adequate dilation of the left lung

Following surgery, bronchoscopic observation of the trachea under anesthesia revealed minimal phlegm. After the patient awoke from anesthesia and began to breathe spontaneously, his pulse oximetry reading was maintained at 100% and he did not complain of postoperative wound pain; thus, the patient was extubated. However, immediately after extubation, labored respiration began and breathing rate markedly increased. Pulse oximetry decreased to 84% under the administration of 6 L/min of oxygen via face mask. There was little sputum or hemosputum, but subcutaneous emphysema was evident in the right subcutaneous area and streak of the large pectoral muscle. The patient exhibited normal breath sounds in the left lung. Further, there was no air leak from the chest tube without kinks, clogs, or regurgitation during inspiration of the chest tube system. The measurements of arterial blood gases were 7.207 for pH, 68 mmHg for PaO_2_, and 64 mmHg for PaCO_2_; accordingly, the condition was diagnosed as hypoventilation-associated hypoxemia/hypercapnia. Hypercapnea cannot be explained by intraoperative opioid use because we did not include opioids for postoperative continuous epidural analgesia. In terms of hemodynamics, the patient’s blood pressure was elevated, and his heart rate had increased. There were no signs of strider or wheeze; additionally, the patient did not complain of pain, was able to breathe deeply as instructed, and exhibited clear consciousness. To improve oxygenation, the use of a bi-level positive airway pressure mask was initiated; however, the patient’s respiratory discomfort remained and his pulse oximetry reading only minimally improved (to 92%). Chest radiography revealed a mediastinal shift, which was suspected to have arisen due to impending tension pneumothorax associated with malfunction of the chest drain (Fig. 1). Thus, suction pressure of the chest drain was increased from − 7 to − 15 cmH_2_O, which immediately improved respiratory discomfort. Chest radiography also revealed improvement of the mediastinal shift and adequate dilation of the left lung (Fig. 2).

Arterial blood gas analysis revealed pH 7.256, PaO_2_ 250.3 mmHg, and PaCO_2_ 60.3 mmHg during the administration of 5 L/min of oxygen via face mask; thus, the patient was transferred out of the operating room. During the administration of 4 L/min of oxygen via face mask on arrival to the intensive care unit (ICU), arterial blood gas parameters were pH 7.352, PaO_2_ 200.3 mmHg, and PaCO_2_ 44.9 mmHg. Because no recurrence of breathing discomfort was observed, the patient was discharged from the ICU the following day. There were no postoperative complications after the patient exited the operating room. The patient was discharged from the hospital on the 16th postoperative day without additional complications.

## Discussion

We encountered a case of respiratory failure, presumably caused by impending tension pneumothorax due to the inflow of air from the chest wall after EPP. Tension pneumothorax (a type of pneumothorax) presents with an abnormal elevation of intrapleural pressure on the affected side, resulting in manifestations including collapse of the affected lung, a low-set diaphragm, mediastinal shift to the unaffected lung, and, when treatment is delayed, reduced cardiac output due to impaired venous return [5, 6]. In this case, there was little circulatory collapse. When tension pneumothorax is left untreated, it may lead to serious conditions, including reduced blood pressure and obstructive shock [5–7]. In cases with tension pneumothorax, an open chest wound or an injury site in the lung exhibits a one-way valve mechanism because it allows air to enter the pleural cavity during inspiration but closes during expiration, thereby leading to the progressive accumulation of air in the pleural cavity. When these conditions persist, the intrapleural pressure on the affected side gradually increases, resulting in the development of tension pneumothorax. If not detected promptly, the condition may trigger cardiac arrest [5, 6]. In general, tension pneumothorax is often worsened by positive pressure ventilation [5, 6]. In the present case, no apparent air leakage was recognized during positive pressure ventilation, indicating no bronchopleural air leakage. Thus, the patient’s respiratory status did not worsen during positive pressure ventilation. However, as spontaneous respiration increased, intrapleural pressure became a strong negative pressure that surpassed the chest drain suction pressure of − 7 cmH_2_O. Additionally, the wound in the chest wall functioned as an antireflux check valve, leading to the development of impending tension pneumothorax [5, 6]. Cardiac herniation is a serious postoperative complication of EPP and is reportedly preventable by avoiding mechanical ventilation, hyperinflation of the remaining lung, coughing on extubation, and excessive suction within the chest drainage tubes [8–10]. As noted above, this spontaneous respiration conflicted with the chest suction pressure because we had chosen a lower pressure (− 7 cmH_2_O) than the setting typically used for postoperative drainage (− 15 cmH_2_O). Treatment of tension pneumothorax is undertaken to remove excess air from the pleural cavity. In the present case, because a chest tube had already been inserted, we increased its suction pressure to the typical level of − 15 cmH_2_O. It is necessary to distinguish impending tension pneumothorax from the occurrence of bronchopleural fistula (BPF). BPF is an air leak from the bronchi due to the rupture of sutures after lobectomy, pneumonectomy, or EPP. BPF has been reported to occur in 1.9% of cases of EPP [11]. BPF and dyspnea appear in close succession because pleural effusion of the affected lung is aspirated to the unaffected lung. Diagnostic characteristics of BPF include a large quantity of sputum or hemosputum, as well as progressive subcutaneous emphysema [12–14]. In addition, BPF is suspected of causing a sudden increase of air leakage from the chest tube. Chest radiography and bronchoscopy are helpful in confirming a diagnosis of BPF [12–14]. First, a chest drain is inserted. Then, closure of BPF is needed in many cases [12–14]. In this case, there was little sputum, hemosputum, subcutaneous emphysema, or air leak from the chest tube. Therefore, the possibility of BPF was excluded.

Other differential diagnoses should include post-pneumonectomy syndrome, which is a complication reported to occur in 1 in 640 pneumonectomy patients [15–17]. Notably, the post-pneumonectomy syndrome is a condition that is characterized by respiratory difficulty triggered by a marked mediastinal shift, which pulls the trachea/bronchus and pulmonary artery, causing them to become compressed and narrowed by the vertebral body or the aorta [15–17]. In the present case, no wheezing originating from the narrowing of the trachea/bronchus was heard; therefore, the possibility of post-pneumonectomy syndrome was excluded.

### Conclusions

The chest drain suction pressure after EPP is often set lower than the typical setting to prevent cardiac herniation as a postoperative complication. In such cases, tension pneumothorax may develop on the affected side. This phenomenon can be effectively treated by increasing the suction pressure in the chest drain.

## Abbreviations

BPF: Bronchopleural fistula
ECG: Electrocardiography
EPP: Extrapleural pneumonectomy
ICU: Intensive care unit
SVC: Superior vena cava
TCI: Target-controlled infusion
